# Single cell RNA sequencing and emerging transcriptomic technologies in Alzheimer’s disease cerebral organoids: a mini review

**DOI:** 10.1186/s13041-026-01335-w

**Published:** 2026-07-24

**Authors:** Maizatul Fazilah Abdul Razak, Francis Yew Fu Tieng

**Affiliations:** 1 Faculty of Medicine and Health Sciences, Universiti Malaysia Sarawak, Kota Samarahan, 94300 Sarawak, Malaysia; 2 Department of Clinical Oncology, Faculty of Medicine, Universiti Malaya, 50603 Kuala Lumpur, Malaysia; 3 Department of Clinical Oncology, Universiti Malaya Medical Centre, 50603 Petaling Jaya, Malaysia; 4 Sunway Medical Clinical Research Centre, Sunway Medical Research Ethics Committee (SREC), 47500 Bandar Sunway, Selangor Malaysia

**Keywords:** Alzheimer’s disease, Cerebral organoids, In vitro, Single cell, Next-generation sequencing

## Abstract

Alzheimer’s disease (AD), the most common cause of dementia worldwide, presents a formidable challenge due to its complex, multifactorial etiology, and limited translational success of existing models. Recent advances in stem cell biology have enabled the generation of three-dimensional human cerebral organoids which exhibit multicellular complexity and self-organisation, recapitulating key aspects of fetal brain development in vitro. This mini review highlights the landscape of AD modelling in human cerebral organoids. While studies using single cell RNA sequencing (scRNA-seq) form the core of this review, we also discuss single-nucleus RNA sequencing, computational deconvolution, and spatial approaches as translationally relevant extensions. We categorise current organoid research into distinct model types: (1) familial AD models driven by *APP*, *PSEN1*, and *PSEN2* mutations; (2) sporadic AD models incorporating genetic *APOE* variants and environmental risk factors (serum exposure, pollutants, and viral reactivation); (3) tauopathy models highlighting astrocyte-driven metabolic disruption; (4) models featuring glial-immune interactions; and (5) emerging models of mitochondrial dysfunction as an early disease initiator. Across these categories, single cell technologies reveal critical alterations in lipid metabolism, synaptic programs, and glial-immune crosstalk. Finally, we review methodological innovations—drawing on non-AD developmental organoid studies such as region-specific patterning, morphodynamic control, and enhanced cellular diversity, which offer a roadmap for improving the physiological fidelity of next-generation AD organoid models. Ultimately, integrating these advanced cerebral organoid platforms with single cell technologies offer a powerful toolkit to dissect early disease mechanisms and accelerate the development of personalised therapeutics.

**Graphical abstract:**

**Figure:**
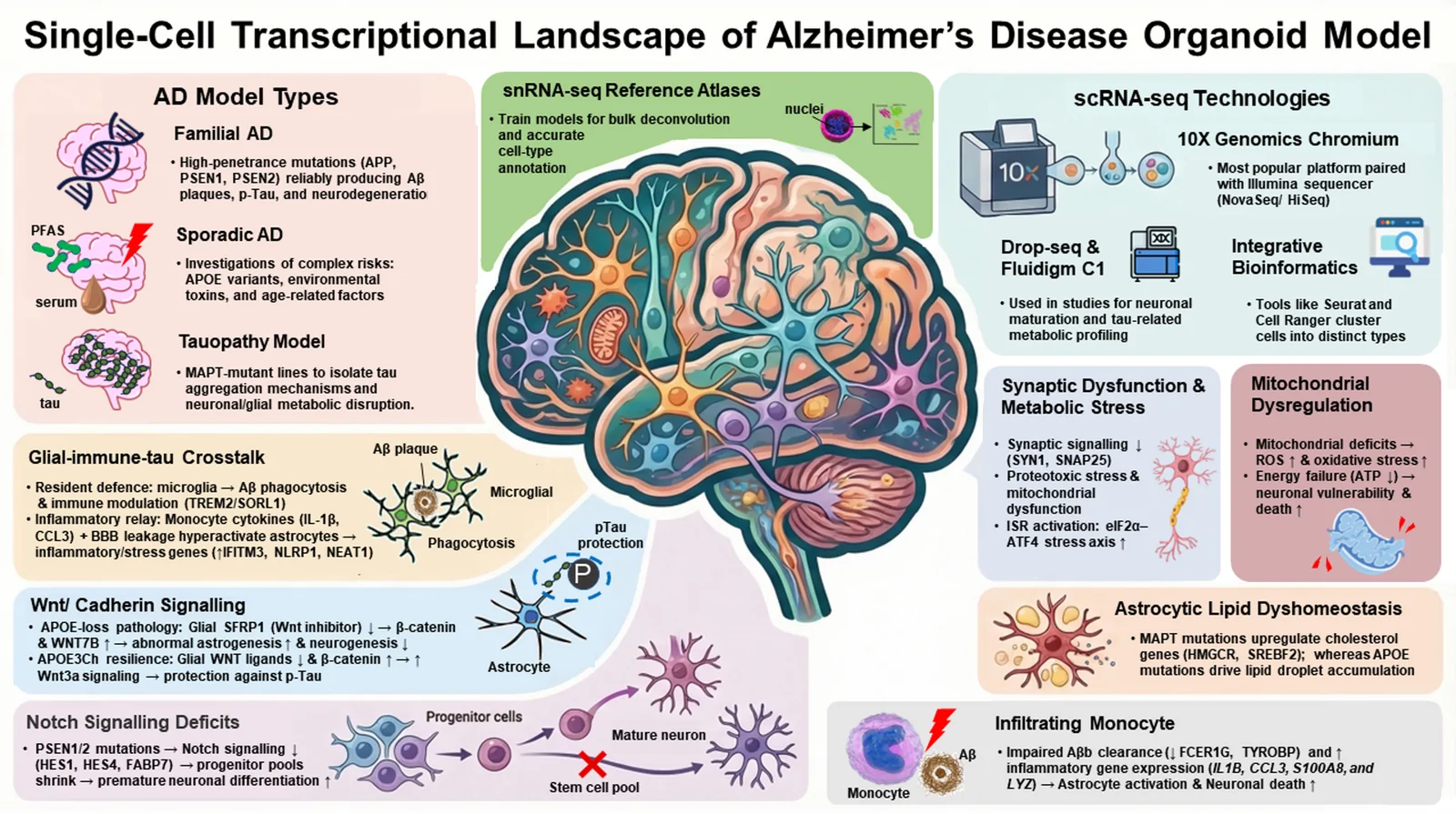
Single-Cell RNA Sequencing Landscape in Alzheimer’s DiseaseOrganoid Models.

**Supplementary Information:**

The online version contains supplementary material available at https://doi.org/10.1186/s13041-026-01335-w.

## Introduction

Alzheimer’s disease (AD) is the most prevalent form of dementia that accounts for 60% to 70% of all cases. It currently affects over 55 million people worldwide, reflecting a 271% increase from 20.3 million in 1990 [16]. This number is expected to surge to 152.8 million by 2050, with the steepest rises projected in low- and middle-income regions, particularly North Africa and Eastern Sub-Saharan Africa [17]. In parallel, the global economic impact of Alzheimer’s and other dementias is estimated to reach $14.5 trillion between 2020 and 2050, equivalent to 0.42% of annual global gross domestic product (GDP) [5]. Despite decades of research, effective treatments remain elusive due to the disease's complex and heterogeneous aetiology. Hallmark features include amyloid-beta (Aβ) plaques, neurofibrillary tangles of hyperphosphorylated tau, neuroinflammation, synaptic dysfunction, and progressive neuronal loss, many of which manifest years before clinical symptoms appear [23, 49]. This underscores an urgent need for experimental models that could capture early-stage AD pathogenesis at the single cell level.

Traditional experimental systems, such as monolayer cell cultures and animal models, have provided accessible platforms for studying AD pathology in the absence of post-mortem or longitudinal human studies [32]. Human neural cell lines are widely used for molecular assays, but they suffer from limited physiological relevance [13]. These immortalised cells, often derived from malignant origins, lack the spatial organisation, cellular diversity, and stable gene expression patterns characteristic of native human brain tissue and may exhibit genetic instability that compromises data interpretation [20]. On the other hand, animal models particularly transgenic mouse models, knock-in/knockout models, and specialised strains like *SAMP8* or seeding models have been instrumental in elucidating key AD hallmarks [55, 62, 67]. Nevertheless, while these models successfully recapitulate many molecular and pathological features of AD, they do not fully reproduce the complexity of sporadic late-onset AD or the human brain's unique developmental trajectory, prolonged aging process, cellular diversity, and regional cytoarchitecture. Furthermore, species-specific differences in gene regulation, neuronal and glial biology, immune responses, and brain connectivity can influence disease manifestation and therapeutic responses, limiting the direct translation of preclinical findings to humans [21, 48].

## Single cell transcriptomic landscape in 3D cerebral organoids

Cerebral organoids are three-dimensional (3D), self-organising tissues derived from human pluripotent stem cells (iPSCs) that have emerged as a valuable human-specific model for studying AD pathology [28]. Unlike other region-specific organoid models, such as midbrain, hypothalamic, or spinal organoids, which represent brain areas less implicated in AD, these miniaturised brain-like structures recapitulate key features of early cortical development, including neuroepithelial organisation, layered neuronal differentiation, and region-specific transcriptional patterning. They also maintain greater cellular diversity and human-relevant gene expression than traditional animal models or immortalised cell lines [35]. In the context of AD, cerebral organoids are particularly relevant due to many early and well-established AD-related pathological processes involving the cerebral cortex and its interaction with cortico-hippocampal networks [12, 26, 31]. Although cerebral organoids might not represent the entire spectrum of AD which notably features early degeneration of the basal forebrain cholinergic systems and eventually impacts widespread brain regions as the disease progresses—they provide an invaluable, human-relevant platform for interrogating the cortical and early network-level mechanisms that are highly relevant to AD [7, 58]. Recent innovations in organoid culture systems, including stem cell optimisation, refined morphogen delivery strategies, modulation of the extracellular matrix (ECM) niche, and manipulation of tissue architecture to better mimic in vivo brain morphogenesis, have significantly advanced the physiological relevance of cerebral organoids [44]. Complementary approaches such as co-culture with microglia-like cells, incorporation of vascular elements, exposure to systemic factors, and the integration of spatio-temporal dynamics have further enhanced cellular heterogeneity, neuronal functionality, and maturation [52].

Single cell RNA sequencing (scRNA-seq) has become indispensable in cerebral organoid research, enabling precise mapping of cell-type diversity, developmental trajectories, molecular states, and protocol-dependent effects, as well as benchmarking against fetal brain tissue and high-throughput screening of disease-associated genes [30, 54]. Unlike bulk RNA sequencing, which averages gene expression across mixed cell populations, scRNA-seq captures transcriptomic signatures at the single cell resolution, making it especially powerful for dissecting subtle but disease-specific cellular heterogeneity [57]. While scRNA-seq has been widely applied to study neurodevelopment, neuroinflammation, and tumourigenesis in cerebral organoids, its application in AD-specific models remains relatively limited but is rapidly expanding [11, 40, 45]. Focusing on this emerging intersection, this mini-review explores how scRNA-seq applied to AD-specific cerebral organoids enables the interrogation of genetic, environmental, immunological, and metabolic processes at the single cell level.

### Familial Alzheimer’s disease: *APP*, *PSEN1* and/or *PSEN2* mutation models

Among the earliest and most well-defined applications of scRNA-seq in AD cerebral organoids has been the modelling of fAD, where autosomal dominant mutations in *APP*, *PSEN1*, or *PSEN2* provide a genetically tractable entry point for dissecting pathogenic cascades. For example, Vanova et al. [58] explored the early impact of *PSEN1* (A246E) and *PSEN2* (N1411) mutations in iPSCs-derived cerebral organoids from fAD patients and isogenic controls. By integrating multi-omics tools, including scRNA-seq, mass spectrometry, immunohistochemistry, and pseudotime trajectory analysis, the study identified not only classical AD-like features such as elevated Aβ_42/40_ ratios and tau phosphorylation (pSer202/pThr205), but also unexpected developmental abnormalities, such as defective tissue patterning, premature neuronal differentiation, and reduced retinal-like zones. scRNA-seq analyses revealed disrupted progenitor-to-neuron transitions and premature neuron development, linked to downregulation of Notch pathway genes (*HES1*, *HES4*, *FABP7*), implicating an early failure to maintain progenitor pools. These findings show that fAD mutations may impair brain development long before symptomatic onset, suggesting early developmental perturbations may contribute to AD vulnerability [58].

Following this, Choe and the co-investigators advanced the field by developing a more mature, and neuropathologically robust fAD model. Pathogenic mutations in *APP* and *PSEN1* were introduced into human pluripotent stem cells, which were then differentiated into cerebral organoids. These isogenic lines exhibited hallmark AD pathologies, including extracellular Aβ plaques, phosphorylated tau (pTau), neurofibrillary tangle–like structures, and neurodegeneration. ScRNA-seq revealed diverse neural and non-neural populations, with transcriptional signatures reflecting disease progression. Treatment with β- and γ-secretase inhibitors ameliorated Aβ and tau pathology, underscoring the model’s translational potential for preclinical drug screening [8].

Unlike earlier efforts that primarily modelled fAD using defined mutations and static time points, the integration of longitudinal single cell data enhances the ability of cerebral organoids to capture dynamic transcriptional changes, cell fate disruptions, and glial dysfunction which are features increasingly recognised in the pathophysiology of sAD.

### Sporadic Alzheimer’s disease: *APOE* genotypes and environmental factors

#### *APOE*-linked genetic risk and resilience

While fAD models leverage deterministic mutations in *APP* and *PSEN* genes, sAD, which accounts for over 95% of cases arises from complex interactions among common genetic risk variants and environmental influences [39]. Among these, *APOE* polymorphisms stand as the most significant genetic modifiers of AD susceptibility [9]. In 2023, Zhao and co-researchers demonstrated that *APOE* knockout in cerebral organoids impairs excitatory neuron development and disrupts lipid metabolism through the activation of the eukaryotic initiation factor 2 (*EIF2*)-mediated integrated stress response (ISR). Using scRNA-seq on isogenic iPSC-derived organoids, they found that APOE deficiency led to altered cell-type composition, reduced neuronal maturity, and upregulation of *activating transcription factor 4 (ATF4)* target genes in neurons. Dysregulated cholesterol efflux and intracellular lipid accumulation further implicated *APOE*’s metabolic role in early-stage AD pathology. Notably, treatment with the ISR inhibitor partially restored neuronal differentiation and mitigated stress-related transcriptomic signatures, identifying the ISR as a modifiable therapeutic axis [66].

Building on the role of *APOE* in disease modulation, Pérez-Corredor et al*.* investigated a rare case of genetic resilience using iPSC-derived cerebral organoids from an individual carrying both a *PSEN1* fAD mutation and the *APOE3 Christchurch* (*APOE3Ch*) variant, who remained cognitively intact into his seventies. Their scRNA-seq and transcriptional network analyses revealed that *APOE3Ch* organoids exhibited enhanced Wnt/β-catenin signalling in both neurons and astrocytes, which are the pathways critical for neuronal differentiation, synaptic stability, and glial homeostasis. Interestingly, this correlated with significantly lower levels of pTau, suggesting that Wnt pathway enhancement may underlie *APOE3Ch*-mediated protection from tauopathy. These protective effects were further validated using isogenic *APOE3Ch*-edited lines, reinforcing the utility of cerebral organoids and single cell technologies in unraveling mechanistic insights into genetic resilience in AD [47].

#### Serum exposure, pollutants, and latent viral infection lead to sAD

Beyond genetic risk, scRNA-seq–based cerebral organoid models have also illuminated how systemic environmental stressors contribute to sAD pathology. For instance, Chen et al*.* established a non-genetic sAD model by exposing human iPSC-derived cortical organoids to human serum, mimicking blood–brain barrier (BBB) breakdown. This triggered hallmark AD features, including Aβ accumulation, pTau elevation, synaptic loss, and neuronal apoptosis. scRNA-seq revealed cell–type–specific transcriptional responses, with both neurons and astrocytes showing downregulation of synaptic genes (*SYN1, SNAP25*) and astrocytes exhibiting an upregulation of inflammatory transcripts (*IFITM3, NLRP1, MT2A*). Serum exposure increased *BACE1* and *GSK3*α/β levels, which in turn promoted Aβ and pTau pathology. However, inhibition of these pathways failed to reverse synaptic degeneration, suggesting the existence of multifactorial disease mechanisms [7]. Additionally, the observed transcriptomic alterations closely mirrored those found in postmortem Alzheimer’s disease cortical tissue, thereby reinforcing the physiological relevance and translational validity of the model [22].

Expanding on environmental modelling, researchers from Sichuan University, China, investigated per- and polyfluoroalkyl substances (PFAS) exposure on human cerebral organoids. Chronic exposure to PFOA, PFOS, and PFHxS led to neuronal loss, increased Aβ and pTau, and disruptions in lipid metabolism. These effects were supported by integrated scRNA-seq and lipidomic analyses, highlighting lipid dysregulation as a key mediator of environmentally induced AD-like neurodegeneration [34].

On the other hand, Feng et al. employed an integrated two-part single cell resolution strategy to investigate how human herpes simplex virus (HSV) infection drives transposable element (TE) dysregulation in AD. Single-nucleus RNA sequencing of post-mortem AD brains from the ROS/MAP and MSBB cohorts revealed widespread TE dysregulation, notably astrocyte-specific upregulation of *LINE1* elements in HHV-positive samples. To validate these findings, the authors analysed existing bulk and scRNA-seq datasets from HSV type1–infected human iPSC-derived forebrain organoids, which showed similar astrocyte-specific *LINE1* activation that was partially reversed by antiviral treatment with valacyclovir or acyclovir. Valacyclovir also rescued tau-associated neuropathology, linking HHV infection, TE activation, and tau pathology in sAD [14]. Notably, while the study integrates post-mortem snRNA-seq with organoid-based scRNA-seq models, these modalities differ substantially in transcriptomic composition, and scRNA-seq datasets from live organoid systems are not directly comparable to snRNA-seq profiles from post-mortem tissue due to weak cross-modality correlations and fundamental biological differences [6, 24]. This limitation should be considered when interpreting cross-system concordance in AD-associated HSV-TE mechanisms.

Despite such inherent methodological limitations, these environmental models illustrate how diverse insults, namely serum factors, pollutants, and latent viruses, can initiate AD-like pathology through converging molecular pathways, including synaptic dysfunction, lipid imbalance, and tau accumulation. These sporadic triggers recapitulate molecular pathways similarly implicated in familial tauopathies, suggesting a potential pathogenic framework underlying both sporadic and familial forms of the disease.

### Tauopathy and developmental models: insight into cell-type pathways

Building on insights from fAD and sAD models, recent efforts have shifted toward modelling tau pathology, a hallmark more closely linked to neurodegeneration and cognitive decline [29]. Tauopathies, whether familial and sporadic, converge on pathological tau aggregation as a shared endpoint [63]. When combined with scRNA-seq, cerebral organoid models enable high-resolution mapping of tau-associated cellular vulnerabilities and developmental trajectories in the human brain. To demonstrate this, Fiock et al. [15] profiled tau expression during cortical development and observed its dynamic upregulation in maturing excitatory neurons. By comparing cerebral organoids with human fetal brain tissue, they validated organoids as a relevant model for studying early tau regulation in neurodevelopment [15].

In contrast, Glasauer et al. developed a familial tauopathy model (specifically modelling frontotemporal dementia) using human cerebral organoids harboring *MAPT* mutations (*V337M* and *R406W*). By employing isogenic iPSC lines with both heterozygous and homozygous variants, they captured gene dosage effects across a developmental time-course (4–8 months). Combination of scRNA-seq and lipidomics revealed early, astrocyte-specific upregulation of cholesterol biosynthesis genes (*HMGCR, ACAT2, STARD4, LDLR,* and *SREBF2*). While astrocytes exhibited metabolic dysfunction, pyramidal neurons showed a coordinated downregulation of glycolysis-related genes (*PGK1, ENO2*) and GABA receptor components, suggesting both cell-autonomous and non-cell-autonomous effects of tau mutations [19].

By juxtaposing findings from Glasauer et al. and Lu et al. (from Sect. 3.2.2), it becomes evident that astrocytic lipid dysregulation emerges downstream of tau pathology in both sporadic (environmental toxicant-induced) and familial tauopathies (genetic *MAPT* mutation-driven, such as frontotemporal dementia). Despite distinct upstream triggers, both models converge on common astrocyte-mediated metabolic disruptions, defining a shared mechanistic axis in tau-driven neurodegeneration across distinct disease classifications [19, 34].

#### Glial-immune-tau crosstalk: a frontier for cerebral organoid models

Glial pathology is increasingly recognised as a key driver of Alzheimer’s disease. Astrocyte-mediated lipid dysregulation and tau toxicity, unfolding within an immune-competent microenvironment, underscore critical pathological mechanisms in AD. Nevertheless, no cerebral organoid models to date have recapitulated tauopathy within a fully integrated immune context. To address this gap, Cakir et al. leveraged cortical organoid by introducing microglia-like cells via the transcription factor *PU.1* overexpression, thereby enabling the study of innate immune interactions. These cells exhibited key morphological and transcriptomic features of native microglia, including the capacity to phagocytose Aβ aggregates. Using CRISPR interference and scRNA-seq, the authors performed functional screening of AD-associated genes such as *TREM2* and *SORL1*, elucidating their roles in modulating Aβ toxicity. This microglia-containing organoid platform provided a tractable system for examining glia–neuron crosstalk and revealed both protective and dysregulated immune responses in early AD-like pathology [3].

Similarly, Tian et al*.* developed a novel and scalable human cortical organoid microphysiological system (hCO-MPS) to assess peripheral immune contributions to AD. This platform integrates 3D-printed scaffolds into a 96-well format to support high-throughput culture of viable, doughnut-shaped cortical organoids. Co-culture with AD patient–derived monocytes revealed increased infiltration, reduced Aβ clearance, and elevated *IL1B* and *CCL3* secretions, inducing astrocyte activation and neuronal apoptosis. scRNA-seq further identified transcriptional states associated with oxidative stress, glial reactivity, and neuronal death, implicating peripheral immune cells as active drivers of neuroinflammation and neural injury in AD [56].

### Mitochondrial dysregulation as AD initiators: a paradigm shift

While Aβ and tau aggregation dominate the landscape of AD research, accumulating evidence implicates that mitochondrial dysregulation may act as an early contributing factor of AD rather than a downstream consequence. In this emerging view, mitochondrial impairment is not merely a downstream consequence of amyloid or tau pathology, but a central orchestrators of early AD-like pathogenic events [1, 4, 38]. This finding aligns with the mitochondrial cascade hypothesis, which states that inherited or acquired mitochondrial deficits initiate oxidative stress and drive sAD pathology [68, 69].

Supporting this model, Pérez et al. use pitrilysin metallopeptidase 1 (*PITRM1*) knockout iPSC-derived cerebral organoids to demonstrate that loss of mitochondrial protease induces widespread proteostatic stress, along with hallmark AD pathologies including Aβ and tau accumulation, and neuronal death. Notably, scRNA-seq further revealed cell-type–specific responses, including astrocytic immune activation, highlighting mitochondrial dysfunction as a driver of early, cell-intrinsic AD features [46]. This indicates that organoids offer a uniquely human, multicellular system to dissect these processes at single cell resolution. As such, they represent a critical platform for future investigations into the initiating role of mitochondrial dysfunction in AD.

## Methodological innovations shaping future cerebral organoids for AD research

Building on evidence that mitochondrial dysfunction, tau pathology, and glial–immune crosstalk are central to early AD processes, cerebral organoids provide a promising platform to model these mechanisms in a physiologically relevant, three-dimensional context that captures aspects of early brain development and cell-type diversity [3, 46, 56]. However, the next leap in AD modelling cannot rely solely on disease-specific studies,it requires adopting breakthroughs from fundamental developmental biology. Current cerebral organoid models are limited by incomplete brain regionalisation, a lack of immune components, and difficulties achieving robust, cell-type–resolved analysis [33, 50, 59]. Recent biological and computational innovations are expanding what cerebral organoids can model, particularly in terms of regional identity, neuronal subtype diversity, and morphogenetic precision. These advances hold particular promise for improving the accuracy and scalability of AD-specific organoid models. For instance, Bertacchi et al. demonstrated that *FGF8* signalling can induce co-development of telencephalic and mesencephalic domains in cerebral organoids derived from human iPSCs. Within these spatially distinct regions, *FGF8* modulates the balance of GABAergic and glutamatergic neurons, alters spontaneous network activity, and regulates genes linked to neurodevelopmental disorders. These findings not only enhance region-specific patterning but also support the generation of multi-regional cerebral organoids suitable for studying circuit-level AD dysfunctions, such as excitatory–inhibitory imbalance and anterior–posterior vulnerability gradients, which are phenomena implicated in selective neuronal susceptibility in AD [2].

Although AD research has traditionally prioritises cortical regions, increasing clinical evidence suggests that AD is a multi-systemic disorder, where non-cognitive symptoms such as weight dysregulation, severe sleep/circadian disturbances, and hypothalamic-pituitary axis disruption often emerge before memory impairment [36, 64]. These early symptoms are driven by early-stage vulnerability in the hypothalamus and its dopaminergic circuits [27, 43]. To provide a platform for capturing these often-overlooked early disease stages, Sarrafha et al. developed a blueprint for creating hypothalamus organoids enriched in diverse dopaminergic neuron subtypes. Through dual-SMAD and *WNT* inhibition alongside *SHH* activation, their protocol achieves robust neurochemical subtype diversity. Importantly, their scRNA-seq analysis revealed significant metabolic reprogramming within these dopaminergic populations, including upregulated shifts in glycolysis and oxidative phosphorylation alongside a targeted downregulation of cholesterol biosynthesis [53]. Because these exact metabolic and lipid pathways are increasingly implicated as core drivers of AD pathology across cortical models [60, 61], adopting this targeted hypothalamic protocol provides the precise methodological framework needed to model the earliest systemic symptoms and region-specific metabolic vulnerabilities of AD.

These region- and subtype-specific innovations are further strengthened by the work of [25], who introduced a live imaging platform to capture the morphodynamics of cerebral organoid development, adding a biophysical dimension to these patterning strategies. Using light-sheet microscopy and fluorescently labeled hiPSCs, they showed that extracellular matrix (ECM) cues and mechanosensitive signalling (e.g., *WNT*, *YAP1*) govern lumen formation, telencephalic expansion, and caudal identity suppression. Their results underscore the importance of biophysical context in shaping organoid morphology and regional fate that provides a scalable, ECM-guided strategy to improve telencephalic fidelity in cerebral organoids used for AD modelling [25].

Finally, on the computational front, tools such as scRNA-seq–informed bulk RNA deconvolution now enable cell-type–specific transcriptomic analysis even when full single cell profiling is impractical [10]. These approaches are essential for connecting disease-relevant phenotypes, such as microglial activation, astrocyte reactivity, and/or neuronal stress response, to specific cellular compartments within heterogeneous organoid models. Complementary methods, including single-nucleus RNA sequencing and spatial transcriptomics, will further enhance the resolution and interpretability of these complex systems.

Together, these advances represent a next-generation toolkit for cerebral organoid-based AD research: combining molecular patterning, neurochemical enrichment, morphodynamic control, and computational resolution to create human brain models that are not only more regionally accurate and functionally relevant but also increasingly scalable for mechanistic and translational studies in AD.

**Fig. 1:**
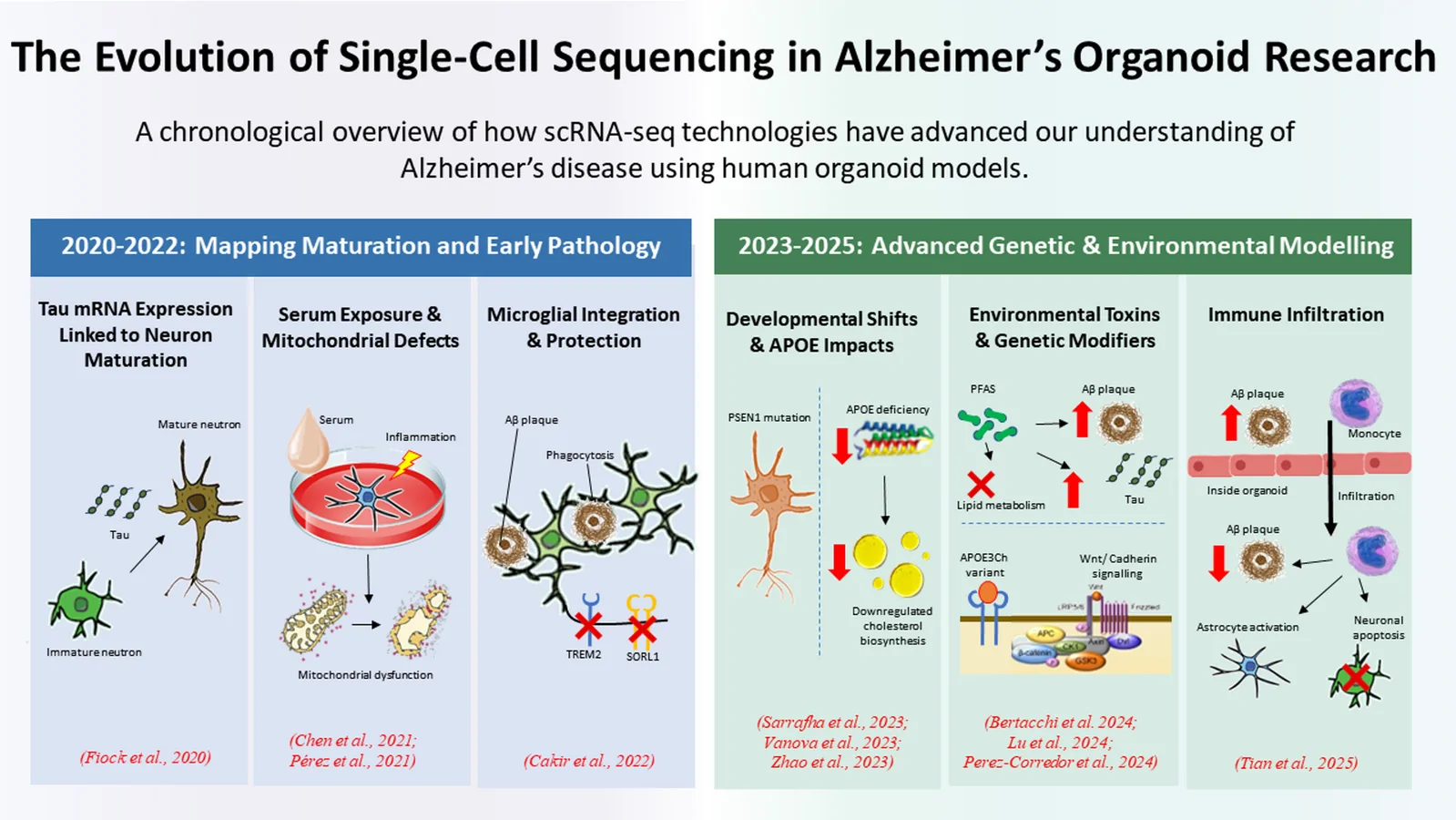
Chronological Overview of scRNA-seq in AD Organoid Research. The timeline illustrates the rapid evolution of scRNA-seq in AD organoid models. 2020–2022: studies established baseline cellular maturation and early disease mechanisms, including tau-associated neuronal maturation, sporadic AD induction via serum exposure and mitochondrial dysfunction, and neuroprotective effects of microglial integration. 2023–2025: advances enabled sophisticated genetic, environmental, and multicellular modelling, revealing the effects of familial AD mutations, APOE deficiency, PFAS exposure, APOE3 Christchurch, Wnt/Cadherin signalling, and monocyte-mediated neuroinflammation on neuronal, glial, and lipid pathways

To capture the rapid evolution of this niche field, Table 1 presents a comprehensive summary of the currently available literature utilising scRNA-seq in human AD-specific cerebral organoids. Rather than selectively sampling the literature, this table outlines the entire current landscape of familial, sporadic, and tauopathy AD organoid models profiled at single cell resolution. Furthermore, a highly selective subset of non-AD organoid studies are included to address current limitations in disease modelling. The inclusion of these non-AD studies is specifically justified by their introduction of critical methodological innovations—such as enhanced regional patterning, morphodynamic control via extracellular matrix cues, and computational bulk deconvolution techniques—which provide a direct roadmap for the next generation of physiologically accurate AD models. Complementing this, Fig. 1 provides a chronological overview of these landmark studies, illustrating the rapid evolution and expanding complexity of scRNA-seq applications in AD organoid research over time.

**Table 1:** Summary of single cell transcriptomic studies in cerebral organoids: Applications, cell type, methodologies, and key findings in models of Alzheimer's disease, tauopathies, and neurodevelopment

Type	Role of scRNA-seq	Type/ Number of cells analysed	Technology & sequencer^a^	Take home messages	Refs
fAD	Analysis of the specific cell types within the CO, revealing a heterogeneous composition of normal brain cells	70-day-old *CAG-APPSweFlLon-IRES-PSEN1M146L/L286V (*CAP) cerebral organoids; cell number not stated	Chromium (10 × Genomics); sequencer not stated	Overexpression of familial AD genes accelerates Aβ and tau pathologies alongside increased apoptosis Pathologies are successfully mitigated by β- and γ-secretase inhibitors, validating the model for preclinical drug screening	[8]
fAD	Identification of a heterogenous mixture of cells Identification of developmental trajectories, showing that clusters enriched in APOE3Ch cells represented later stages, suggesting altered developmental timing or premature differentiation Identification of regulation of Cadherin and Wnt signalling pathways by APOE3Ch	6,400 cells from 6 pooled organoids per iPSC-derived cell line (total 8 lines from 2 patients, α and ω): α-APOE3/PSEN1 E280A, α-APOE3Ch/PSEN1 E280A, α-APOE3Ch/PSEN1WT, α-APOE3Ch/PSEN1 E280A (duplicate clone), ω-APOE3/PSEN1 E280A, ω-APOE3/PSEN1 E280A (duplicate clone), ω-APOE3/PSEN1WT, ω-APOE3/PSEN1 E280A	Chromium (10 × Genomics); sequencer not stated	APOE3Ch expression shifts developmental timing and alters Wnt/Cadherin signalling Enhanced β-catenin offers PSEN1-independent protection against tauopathy	[47]
fAD	Identification of heterogeneity of NDC#1- and AD#1-iPSC-derived organoids Confirmation of the altered developmental phenotype and signs of premature onset of differentiation in a subset of neurons via integrated data analysis (NDC#1-CO and AD#1-CO)	2,298 NDC cells and 2,795 AD cells retained post-QC; derived from 18 pooled day-60 organoids per sample	Chromium (10 × Genomics); NextSeq 500 (Illumina)	PSEN1/2 mutations induce early amyloid/tau accumulation and neurodegeneration Reduced Notch signalling causes early progenitor pool depletion and premature neuronal differentiation	[58]
Aβ-induced AD	Integration of organoid-derived microglia-like cells (oMG) and primary microglia signatures to assess differences between innate oMG and PU.1-induced microglia Identification of developmental trajectories, segregating cells into microglia, neurons, astrocytes, and other lineages to trace lineage commitment (linking GFP/PU.1 expression to microglial fate) Evaluation of the transcriptional response and protective role of microglia following Aβ_1−42_ exposure Analysis of high-throughput genetic perturbations (CROP-seq) to identify the specific roles of AD-associated risk genes (e.g., *SORL1*, *TREM2*) in cholesterol metabolism and Aβ response	13,416 hCO cells and 13,546 mhCO cells for baseline characterisation; 49,867 cells analysed for the non-treated vs. Aβ-treated comparison	Chromium (10 × Genomics); NovaSeq S4 (Illumina)	PU.1-induced microglia provide a neuroprotective, phagocytic response to Aβ toxicity This immune protection is critically dependent on AD risk genes like *TREM2* and *SORL1*	[3]
sAD/ immune-driven	Investigation of mechanisms of increased AD monocyte infiltration into the human cortical organoid microphysiological systems (hCO-MPSs) Analysis of neuronal and monocyte interactions in the hCO-MPS model to reveal immune-driven neurodegeneration	Cells from hCO-MPSs that were cocultured with human peripheral monocytes; number not stated	Chromium (10 × Genomics); sequencer not stated	Infiltrating AD-derived peripheral monocytes show impaired Aβ clearance Monocytes drive astrocyte reactivity and neuronal apoptosis via IL-1β and CCL3 inflammatory pathways	[56]
sAD/ viral induced	Profiling of transcriptomes from 60-day-old organoids at 3 days post infection and clustering with distinct cell types Mechanistic validation of snRNA-seq, pinpointing cell types in the HSV-1/HHV-infected human organoid model that drive TE dysregulation and to assess how antiviral drugs modulate these effects Identification of cell type-specific transcriptional changes in transposable elements (TEs) and genes following HHV infection Investigation of the underlying mechanisms of acyclovir and valacyclovir on HSV-1-infected human brain organoids to assess how antiviral drugs modulate TE dysregulation	Three public datasets: scRNA-seq (GSE129308): 24,660 soma with NFTs, 38,465 neighbouring NFT-free neurons from AD donors and 63,110 control NFT-free somas snRNA-seq (GSE138852): 13,214 nuclei of entorhinal cortex from control and AD brains of twelve individuals snRNA-seq (GSE148822): 482,472 nuclei from non-demented control brains and AD brains containing only amyloid-b plaques or both amyloid-b plaques and tau pathology Validation = droplet-based scRNA-seq public dataset (GSE163952): human iPSC-derived organoid cells; number not stated	GSE129308 [42]: Chromium (10 × Genomics); NovaSeq 6000 (Illumina) GSE138852 [22] & GSE148822 [18]: Chromium (10 × Genomics; NextSeq 500 (Illumina) Validation scRNA-seq public datasets: GSE163952 [51]: Drop-seq (ChemGenes Corp.); NextSeq 500 (Illumina)	Latent human herpesvirus (HHV) induces cell-type-specific (notably astrocyte and microglia) transposable element (LINE-1) activation Antiviral treatment (valacyclovir/acyclovir) partially rescues both LINE-1 activation and downstream tau pathology	[14]
sAD/ environmental induced	Prediction and validation of cell-type-specific neurotoxicity of PFASs A powerful methodology to evaluate the health risks associated with environmental contaminants, particularly those with neurotoxic potential	4 organoids per group (control and 900 × PFAS): 14,556 cells from control group and 14,030 cells from 900 × PFAS group	Chromium (10 × Genomics); NovaSeq X Plus (Illumina)	Chronic PFAS exposure triggers AD-like neurodegeneration, Aβ/pTau accumulation, and synaptic loss Pathology is heavily mediated by severe dysregulation of glycerophospholipid and sphingolipid metabolism	[34]
sAD	Elucidation of the cell-type-specific effects of APOE deficiency in human cerebral organoids Identification of changes in cellular composition, demonstrating that excitatory neurons were reduced while astrocytes, radial glia, and inhibitory neurons increased	27,199 single cells in total (14,920 cells from control Day-90 cerebral organoids and 12,279 cells from isogenic APOE-/- Day-90 cerebral organoids)	Chromium (10 × Genomics); HiSeq 4000 (Illumina)	APOE deficiency impairs excitatory neurogenesis and triggers astrocyte proliferation via Wnt signalling Activation of the EIF2-integrated stress response and disruption of lipid/cholesterol homeostasis, a phenotype mirrored by the APOE4 genotype	[66]
sAD	Identification of a heterogenous mixture of cells Evaluation of synaptic and immune transcriptomic alterations in serum-exposed sAD organoids to reveal reduced synaptic function in neurons and induced immune responses in astrocytes	26,631 cells (9,338 BO2_control; 5,412 BO2_serum; 6,535 BO3_control; 5,346 BO3_serum)	Chromium (10 × Genomics); NovaSeq 6000 (Illumina)	Serum exposure (mimicking blood–brain barrier breakdown) drives parallel progression of Aβ, pTau, and astrocyte inflammation Synaptic decline is not reversed by BACE1 or GSK3 inhibitors, indicating multifactorial disease mechanisms	[7]
Tauopathy/ familial	Profiling of human cerebral organoids from MAPT-mutant lines vs isogenic controls across development Identification of a heterogenous cell population Identification of cell-type-specific pathway changes, specifically uncovering cholesterol biosynthesis dysregulation in astrocytes and glycolysis/synaptic drops in neurons Nomination of disease-relevant mechanisms downstream of tau mutations	76,111 total cells post-filtering, with a mean of 1,228 cells ± 239.6 SD per sample	Drop-seq [37]; NextSeq 500 (Illumina)	Familial MAPT mutations drive a progressive, APOE-independent overactivation of cholesterol biosynthesis in astrocytes Pyramidal neurons exhibit a coordinated downregulation of glycolysis and synaptic components	[19]
Tauopathy/ developmental	• Evaluation of tau (MAPT) mRNA expression patterns as a function of lamina and neuronal maturation during fetal brain development and in iPSC-derived cortical organoid • Confirmation of low amounts of tau mRNA in very early neuronal precursors (radial glia and intermediate progenitors)	2 public datasets: • 48 human fetal cortex samples aged 5.8–37 postconceptional weeks; number not stated [41] • hiPSC-derived cortical organoids derived from three different donors at 105 day of in vitro differentiation; number not stated [65]	• Fluidigm C1 (Fluidigm) and HiSeq2500 (Illumina) [41] • BD Rhapsody (BD Biosciences) [65]	Organoids replicate the spatiotemporal upregulation of tau observed during human foetal brain development Human foetal brain and organoids share a conserved tau-upregulation trajectory during neuronal maturation—useful as a baseline for disease models	[15]
Familial risk mitochondrial dysfunction	Characterisation of cell-type-specific pathogenetic mechanisms, mitochondrial dysfunction, and AD-like pathology in PITRM1-deficient organoids	Total 15,183 cells from 2-month-old cerebral organoids (WT + KO pooled; per-genotype counts not reported; ~ 18,000 cells loaded per sample pre-QC)	Chromium (10 × Genomics); sequencer not stated	PITRM1 knockout shows that primary mitochondrial dysfunction initiates widespread proteotoxic stress Knockout organoids drive early AD-like Aβ and tau pathology that can be partially rescued by NAD + precursors (NMN)	Pérez et al. [46]
Developmental/regional modelling (neurodevelopment disorder)	Investigation of the transcriptomic signature of cerebral organoids upon FGF8 treatment to quantify regional and neuronal subtype diversity shifts, illuminating mechanisms of regional vulnerability	18,590 cells analysed from Day 69 organoids (from two independent batches per condition [WNTi; WNTi + FGF8], each batch pooling 2–3 organoids)	Chromium (10 × Genomics); sequencer not stated	FGF8 signalling induces the co-development of distinct telencephalic and mesencephalic domains A multi-regional model suitable for studying selective vulnerability and circuit-level dynamics	[2]
Non-AD (developmental modelling/ Parkinson’s disease)	Characterisation of neuroendocrine and dopaminergic diversity in hypothalamic organoids Identification of molecularly distinct hypothalamic DN subtypes and their respective developmental maturities	21,001 cells from D85 hypothalamus organoids (TH^hi^ group: 10,464 cells, ≈2,647–4,015 per replicate; TH^lo^ group: 10,537 cells, ≈3,262–3,663 per replicate)	Chromium (10 × Genomics); sequencer not stated	Guided patterning yields 7 distinct dopaminergic neuron (DN) subtypes with varying developmental maturities DNs exhibit profound metabolic reprogramming, including upregulated glycolysis and downregulated cholesterol biosynthesis	[53]
Non-AD (developmental modelling	Mapping of regionalisation and morphogenetic transitions in unguided brain organoids Evaluation of cell-state changes associated with tissue transitions and the molecular processes affected by different extracellular matrix (ECM) and signalling perturbations	Time-course (D5, D7, D11, D16, D21, D30): Per-day cell counts not reported; pooled organoids = 24 (D5/D7/D11), 12 (D16), 10 (D21), 6 (D30) Day 13 – Matrix: Matrigel 8,095; No-matrix 8,343; Agarose 3,725 Day 10 – YAP (Py-60): DMSO 2,085; Py-60 day-5 763; Py-60 day-7 1,955 Day 55 – Hashed (WT + KO totals): Matrigel 5,221 (3,560 + 1,661); No-matrix 5,377 (1,283 + 4,094); CHIR 5,084 (3,310 + 1,774); Py-60 2,782 (1,399 + 1,383); DMSO 3,540 (2,087 + 1,453) Extended Data (hashed subset): Matrigel 1,183; No-matrix 1,285; Py-60 1,189	Chromium (10 × Genomics); sequencer not stated	Extracellular matrix (ECM) cues govern lumen formation and accelerate telencephalic regional fidelity Mechanosensitive YAP-WNT signalling controls the balance of telencephalic versus caudal patterning	[25]
Non-AD (protocol that support AD studies)	Evaluation of nine existing sample-wise cell deconvolution methods (DSA, OLS, CIBERSORT, dtangle, MuSiC, Bisque, bMIND, swCAM, TCA) on human brain transcriptomic data using matched bulk RNA-seq, sc/snRNA-seq, and IHC data to establish best practices and explore biological applications	Matched datasets used for benchmarking/analysis**:** ROSMAP bulk RNA-seq n = 1112; ROSMAP snRNA-seq 48 donors / 69,611 nuclei; IHC n = 49. CMC bulk n = 572; CMC snRNA-seq 101 donors / 569,289 nuclei. Brain organoids bulk n = 130; organoid scRNA-seq 72 samples / 490,844 cells. Across all, **≈**1,130,767 nuclei/cells from 149 adult brains and 72 organoid samples	snRNA-seq and scRNA-seq (public datasets downloaded from Synapse); specific sequencer and single cell platforms are not stated	Benchmarking identifies dtangle and bMIND as the optimal computational framework for bulk RNA deconvolution Accurate inference of cell-type-specific proportions and expression when single cell profiling is impractical	[10]

^a^Denotes by scRNA-seq platform (brand name) and sequencing platform (brand name)

Aβ = amyloid beta, AD = Alzheimer, s disease, APOE = apolipoprotein E, BACE1 = beta-secretase 1, CCL3 = C–C motif chemokine ligand 3, CO = cerebral organoids, CROP-seq = CRISPR droplet sequencing, DMSO = dimethyl sulfoxide, DN = dopaminergic neuron, ECM = extracellular matrix, EIF2 = eukaryotic initiation factor 2, fAD = familial Alzheimer’s disease, FGF8 = fibroblast growth factor 8, GFP = green fluorescent protein, GSK3 = glycogen synthase kinase 3, hCO = human cortical organoid, hCO-MPSs = human cortical organoid microphysiological systems, HHV = human herpesvirus, hiPSC/iPSC = human induced pluripotent stem cell, HSV-1 = herpes simplex virus type 1, IHC = immunohistochemistry, IL-1β = interleukin-1 beta, KO = knockout, LINE-1 = long interspersed nuclear element-1, MAPT = microtubule-associated protein tau, mhCO = microglia-containing human cortical organoids, NDC = non-demented control, NFTs = neurofibrillary tangles, NMN = nicotinamide mononucleotide, oMG = organoid-derived microglia-like cells, PFAS/PFASs = per- and polyfluoroalkyl substances, PITRM1 = pitrilysin metallopeptidase 1, PSEN1 = presenilin 1, PSEN2 = presenilin 2, pTau = phosphorylated tau, PU.1 = PU box-binding transcription factor, QC = quality control, RNA-seq = RNA sequencing, ROSMAP = Religious Orders Study and Memory and Aging Project, sAD = sporadic Alzheimer’s disease, scRNA-seq = single cell RNA sequencing, SD = standard deviation, snRNA-seq = single-nucleus RNA sequencing, SORL1 = sortilin related receptor 1, TEs = transposable elements, TH = tyrosine hydroxylase, TREM2 = triggering receptor expressed on myeloid cells 2, WT = wild type

## Conclusion

Cerebral organoid models, combined with scRNA-seq, represent a transformative platform for investigating the multifaceted pathogenesis of AD. By enabling single cell resolution of cell-type–specific trajectories, developmental disruptions, and context-specific responses to genetic and environmental insults, these models address key limitations of traditional systems. Recent findings including the upstream role of mitochondrial dysfunction, the convergence of environmental and genetic risk pathways, and the impact of glial–immune interactions underscore the potential of cerebral organoids to capture early, modifiable stages of disease. Moreover, innovations in organoid engineering, brain regionalisation techniques, and computational analysis significantly enhances the scope and fidelity of these models.

Looking ahead, integrating immune competence, metabolic stressors, and tauopathy will be essential for capturing the full spectrum of AD pathology. By combining insights from developmental biology, engineering, and data science, next-generation cerebral organoids hold great promise not only for understanding disease mechanisms but also for accelerating therapeutic discovery.

## Supplementary Information

Additional file1 (DOCX 46 kb)

## Data Availability

No datasets were generated or analysed during the current study.

## Abbreviations

Aβ: Amyloid beta
AD: Alzheimer’s disease
APOE: Apolipoprotein E
APP: Amyloid precursor protein
BACE1: Beta-secretase 1
bMIND: Bayesian Model-based INtegration from deconvolution
CMC: CommonMind consortium
CO: Cerebral organoid
DE: Differential expression
DHC: Dehydrocholesterol
dtangle: Deconvolution method for cell-type heterogeneity
ECM: Extracellular matrix
eQTLs: Expression quantitative trait loci
fAD: Familial Alzheimer’s disease
FGF8: Fibroblast growth factor 8
GWAS: Genome-wide association study
hcO-MPSs: Human cortical organoid–microphysiological systems
HHV: Human herpesvirus
hiPSC: Human induced pluripotent stem cell
hPSC: Human pluripotent stem cell
HSV-1: Herpes simplex virus type 1
IF: Immunofluorescence
IHC: Immunohistochemistry
KO: Knockout
LTP: Long-term potentiation
mhCOs: Microglia-containing human cortical organoids
mRNA: Messenger RNA
NDC: Non-demented control
NFTs: Neurofibrillary tangles
NMN: Nicotinamide mononucleotide
NSCs: Neural stem cells
OC: Occipital cortex
oMG: Organoid-derived microglia-like cells
OPCs: Oligodendrocyte precursor cells
OTC: Occipitotemporal cortex
PFASs: Per- and polyfluoroalkyl substances
PHF-tau: Paired helical filament tau
pTau: Phosphorylated tau
PSEN1: Presenilin 1
PSEN2: Presenilin 2
ROSMAP: Religious Orders Study and Memory and Aging Project
scRNA-seq: Single cell RNA sequencing
SCZ: Schizophrenia
SVZ: Subventricular zone
TEs: Transposable elements
TFs: Transcription factors
VLMCs: Vascular and leptomeningeal cells
VZ: Ventricular zone
WNTi: WNT inhibition
