# Supplementary material for: Single cell RNA sequencing and emerging transcriptomic technologies in Alzheimer’s disease cerebral organoids: a mini review
Source: Mol Brain. 2026 Jul 24;19:72. doi: 10.1186/s13041-026-01335-w (PMC13681954; doi:10.1186/s13041-026-01335-w)
Supplement: Supplementary file 1 [file 13041_2026_1335_MOESM1_ESM.docx]

**Supplementary Table 1: Cell Clusters, Marker Genes, and Detailed Research Findings in Single Cell Transcriptomic Studies of Alzheimer’s Disease Cerebral Organoids**

| **Ref.** | **Cell clusters** | **Genes involved** | **Detailed research findings** |
| --- | --- | --- | --- |
| (Choe et al., 2024) | 17 clusters:   - Stem cells - Radial glial cells - Neuroepithelial cells - Pericytes - Neuronal progenitor cells - Intermediate progenitor cells - Astrocytes - Macroglia - Choroid plexus - Mesenchymal - Endothelial cells - Microglia - Five neuron subtypes | ***APP, PSEN1,* lineage markers** | - **Overexpression → AD-like pathology:** fAD gene overexpression in hPSC-COs boosts Aβ₄₀/₄₂ and PHF-tau vs controls (CAP > CA); abundant amyloid deposits (Amylo-Glo, thioflavin-S, 6E10/4G8) - **Neurodegeneration signals up:** More NFT-like structures and higher CASP3 (apoptosis) - **Drug-responsive model:** BACE1 inhibitor IV or γ-secretase inhibitor (Compound E) lowers Aβ and eases PHF-tau/NFT-like changes → suitable for drug testing - **Maturation/overexpression needed:** 70-day patient-derived hiPSC COs **without** overexpression show no Aβ/PHF-tau increase vs wild-type → possibly need overexpression or longer culture |
| (Perez-Corredor et al., 2024) | 15 clusters with 6 major cell types:   - Neuronal (0, 1, 8, 10, 12, 13, 14) - Neuroblast (2) - Radial glia (3, 5, 7) - Neuro endothelial (4, 11) - Oligodendrocyte (6) - Progenitor (9) | ***PSEN1, APOE3Ch, cadherins, WNT2B, WNT4, WNT7B,  β-catenin, APP, MAPT, RELN*** | - **Shifted development:** APOE3^Ch^ organoids skew toward later cell states (clusters 2–3), indicating altered timing/identity - **Core pathways hit:** **Wnt** and **Cadherin** signalling are the most affected across neurons and glia - **Ligands down, β-catenin up:** Glial **WNT2B/WNT4/WNT7B** transcripts drop; **β-catenin mRNA** is unchanged, but **protein** increases and spreads in rosettes, seen in patient brain tissue - **APOE-driven tau effects:** Together with pTau data, supports a **PSEN1-independent, APOE-dependent** influence on tau-related signalling |
| (Vanova et al., 2023) | 11 clusters with 9 major types from integrated NDC#1-CO and AD#1-CO sequencing data:   - NSCs; radial glia (0, 7) - Immature/mature neurons (1, 5) - Intermediate progenitors; neuroblasts (2) - NSCs; radial glia; choroid plexus (3) - Retinal progenitor cells (4) - Retinal progenitor cells; neural retina (neurons, photoreceptor cells, bipolar cells) (6) - Meningeal cells; glial cells (OPCs, microglia); mesoderm (8) - Forebrain neurons; retinal neurons (9) - Meningeal cells; Mesoderm (10) | *PSEN1, PAX6, SIX3, RAX, CRX, HES1, HES4, HES5, FABP7* | - **Amyloid pathology:** Early Aβ accumulation (elevated Aβ_42/40_ ratio) and increased APP-CTFs; drug-responsive to β/γ-secretase inhibitors - **Neurodegeneration markers:** Elevated phospho-tau and neurofilament proteins; reduced CHAT; increased stress, apoptosis, and senescence - **Developmental trajectory:** Premature neuronal maturation with reduced retinal-like cell populations - **Signalling defects:** Reduced Notch activity in progenitors (reduced HES1/HES4 and FABP7 expression) |
| (Cakir et al., 2022) | 14 annotated cell-type clusters:   - Cortical neurons - Interneurons - Neuron-non-committed - Neural progenitors - Astrocytes - Glial progenitors - BMP-responsive cells - Cilia-bearing cells - Unfolded-protein-response-related cells - Unassigned - Proteoglycan-expressing cells - *C1QA^+^, C1QC^+^, CCL3/4^+^* microglia - *TREM1^+^, NFKB1^+^* microglia precursor A1 - *CXCL1^+^, MYB^+^, IRF8^+^* microglia precursor A2 | ***TREM2, TYROBP, IL1B, TNF, CD68, CX3CR1, P2RY12*** | - **Microglia-like cells in organoid:** PU.1 induction creates mhCOs with bona fide microglia and precursors matching yolk-sac–like and human fetal microglia profiles - **Protective Aβ response:** On Aβ₁–₄₂ exposure, these microglia activate and phagocytose more Aβ, reducing surface apoptosis and easing neuronal injury - **Closer to primary cells:** PU.1-induced microglia resemble primary microglia more than oMG, and Aβ pushes them even further toward this state - **Key genes control protection:** Endocytosis genes (PICALM, SORL1, BIN1) link to cholesterol pathways during Aβ stress. Knocking down **TREM2** or **SORL1** removes microglial protection and increases Aβ-toxicity; **SORL1** loss lowers **CYP46A1** → impaired cholesterol turnover |
| (Tian et al., 2025) | Seven cell types in hcO-MPSs**:**   - Neurons - Astrocytes - Other glial cells - Monocytes - Intermediate progenitors - Progenitors - Others | ***CCR1, SEMA6B, FGR, APLP2, NF1, NUMB, PECAM1, CYP1B1, FOXO3, FCER1G, FCGR2A, TYROBP, GAS6, RAB5A, RAB11A, CLU, LRP2, S100A8, LYZ, ATF4, JUN, GADD45G, ITGB1, RPS3, ANP32A, IL1B, CCL3.*** | - **Stronger entry, weaker cleanup:** AD monocytes infiltrate organoids more than controls (↑ with Aβ), but once inside they **phagocytose less Aβ,** downregulate phagocytosis/Aβ-catabolism genes, upregulate inflammatory genes, as well as triggering **astrocyte activation and neuronal apoptosis** - **Key mediators:** **IL-1β** and **CCL3** are elevated (RNA + protein), supporting a **peripheral monocyte–driven neuroinflammation** pathway in AD |
| (Feng et al., 2025) | Six clusters at occipital cortex region   - Endothelial - Mural cells - Astrocytes - Microglia - Oligodendrocytes - Neurons   Five clusters at occipitotemporal cortex region   - Endothelial - Mural cells - Astrocytes - Microglia - Oligodendrocytes   Four cell types in HSV-1-infected and uninfected organoids:   - Early neuron - Progenitors - Choroid plexus - Mesodermal | ***IRF8, STAT6, TLR2, PPARA, APP, CLU, TGFB2, ITPKB, NEAT1), LPAR1, IL4R, EPHB2, NFAT5, YWHAQ, NRXN1, L1PA10, L1MCa, L1MA5, L1MB3, L1MEd, L1MA9, L1MA2, L1M4c, L2b*** | - **Cell-type TE boost in HHV⁺ AD:** LINE-1 elements rise in astrocytes, microglia, and oligodendrocytes (e.g., L1PA10/L1MCa/L1MA5); microglia also show immune TFs (IRF8) and inflammatory pathways - **Genomic links & staging:** L2b **↔ ITPKB** and L1M4c **↔ NEAT1;** L1M4c tracks Braak stage. **NEAT1** is specifically up in astrocytes across regions (OC/OTC) - **Model + rescue:** HSV-1–infected forebrain organoids replicate LINE-1 upregulation in microglia/astrocytes, and antivirals (valacyclovir/acyclovir) reduce LINE-1 activity and tau-related pathology |
| (Lu et al., 2024) | 18 clusters with 5 major cell types:   - Neural progenitor cells (15, 17) - Intermediate progenitors (8) - Neurons (0, 2, 3, 4, 6, 7, 9, 12, 13, 14) - Astrocytes (1, 16) - Radial glia (5, 10, 11). | ***APP, MAPT, SMPD1, SPTLC1,* oxidative stress genes** | - **Cell mix shifts to glia:** PFAS reduces neurons/radial glia and increases astrocytes. - **AD-like gene programs:** Neurons show mitochondrial stress ↑ and synaptic genes ↓; astrocytes show immune genes ↑ and synapse terms ↓—matching AD cortex - **Pathology + energy hits:** Aβ_40/42_ and pTau rise; lipid droplets build up; mitochondria malfunction - **Lipid pathway link to Aβ/tau:** Lipidomics (517 lipids) shows glycerophospholipid/sphingolipid disruption; 74 lipids ↑ at high dose (900×); gene shifts (SPHK1, SMPD2, BACE1, APH1A) connect sphingolipid changes to Aβ/tau |
| (Zhao et al., 2023) | 19 clusters with 5 major cell types:   - Excitatory neurons (0, 1, 2, 3, 5) - Radial glia (4, 7, 9, 11, 17) - Astrocytes (10, 12, 13) - Intermediate progenitor cells (6) - Inhibitory neurons (8, 14, 15) - Undecided cluster (16, 18) | ***APOE, SFRP1, ABCA1, ABCG1, DCX, SLC17A7, TBR1, BCL11B, SATB2, SOX2, S100B, GFAP, HMGCS, MSMO, HMGCR, IDI1, FDPS, SQLE, FDFT1*** | - **Cell mix shifts:** Fewer excitatory neurons; more astrocytes, radial glia, and inhibitory neurons - **Stress pathway on:** EIF2–ISR is activated in APOE^−/−^ organoids; **ISRIB** reverses it. - **More astrogenesis via Wnt:** APOE loss ↓SFRP1 → ↑Wnt/β-catenin in astrocytes → more astrocytes - **Lipid/cholesterol dysregulation:** Excitatory neurons ↑cholesterol-biosynthesis genes; astrocytes accumulate lipids and ↑ABCA1/ABCG1 - **APOE4 genotype: APOE4 organoids mirror the APOE-/- phenotype (impaired neurogenesis and cholesterol defects) when compared to isogenic APOE3 organoids → supports a loss-of-function mechanism for APOE4** |
| (Chen et al., 2021) | 14 clusters (no clear definition):   - Six neuronal subclusters - Four astrocytes subclusters - Four neuroepithelial subclusters | ***BACE1, GSK3α, GSK3β, HRK, CNR1, GABRB1, GABRG2, GRIA1, GRIA2, LRRC7, LRRTM2, LRRTM4, MT2A, NEAT1, PRDX6, NLRP1, IFITM3, GABRA2, GLUL, GPM6A, NLGN1, NRXN1, PTN*** | - **Serum drives parallel AD-like changes:** Aβ and pTau both increased. BACE1 inhibitor IV lowers **Aβ** (not pTau); GSK3 inhibitor (CHIR99021) lowers **pTau** (not Aβ) → partly independent pathways - **Synapse programs drop; astrocytes inflame:** Neuronal synaptic genes fall (e.g., CNR1, GABA/AMPA components, LRRC7/LRRTM2/4). Astrocytes show stronger immune signatures (e.g., MT2A, NEAT1, PRDX6, NLRP1, IFITM3) and fewer synapse-related terms, echoing AD cortex - **Functional decline not rescued:** After serum, synapses shrink (↓SYN1 puncta) and networks weaken (↓spikes, bursts, firing, synchrony). Neither BACE1 nor GSK3 inhibition restores synapses → multiple parallel pathologies |
| (Glasauer et al., 2022) | 14 neuronal populations:   - Excitatory pyramidal neurons *(*BCL11B, SOX5, TBR1, SATB2, FEZF2, SLCC17A7*)* - GABAergic neurons *(*GAD1, GAD2, DLX1,2,5,6, ETV1, PBX3, ARX, SP9*)* - Inhibitory neurons *(*GAD1, GAD2 but lacking IN-GE markers*)* - Largely unidentified excitatory neurons - Unidentified neurons *(*SYT1, CPLX2, SYNJ2*)* - Neural progenitor cell 1 - Neural progenitor cell 2 - Radial glia *(*VIM, SOX2, PAX6, EMX2*)* - Actively proliferating cells *(*RGC/NPC cycling; VIM, EMX2, DLX1, TOP2A, CENPF*)* - Largely unidentified glia (TTR, HTR2C, TM4SF1, EFNB3, WNT2B, BMP7*)* - Oligodendrocyte progenitors *(*OLIG1, OLIG2, PDGFRA*)* - Astrocytes *(*SLC1A2, GFAP, S100B, AQP4*)* - Glial progenitor cells *(*SLC1A3, GFAP, S100B*)* - Mesenchymal cells *(*LUM, DCN*)* | ***MAPT, HMGCR, ACAT2, STARD4, LDLR, SREBF2, APOE, ALDOA, ALDOC, ENO1, ENO2, HK1, PFKP, PGK1, LDHA, GABRA2, GABRG2, NPAS4*** | - **Astrocyte cholesterol program up:** MAPT-mutant organoids show higher expression of cholesterol genes (e.g., HMGCR, SREBF2), more **HMGCS1⁺** astrocytes, and this **intensifies from 4→8 months** without changing overall cell proportions - **Neuronal metabolism/synapses down:** Pyramidal neurons reduce **glycolysis** genes and **GABA receptor** genes → coordinated neuron–glia metabolic and synaptic disruption - **Progressive lipid imbalance:** By ~7 months, lipidomics shows higher **cholesterol** and late-pathway intermediates (desmosterol, 7-/8-DHC, cholestanol) → ongoing cholesterol dyshomeostasis - **APOE-independent effect:** Astrocyte cholesterol-gene upregulation with **APOE** downregulation occurs **regardless of APOE genotype** tested |
| (Fiock et al., 2020) | Four cell type based on classic neuronal maturation markers:   - GFAP^+^/NES^+^ radial glia - TBR2^+^ intermediate progenitors - TBR1^+^ immature neurons - SYN^+^ mature neurons | ***MAPT, TBR1, SYN, GFAP, NES, EOMES (TBR2), SYP*.** | - **Tau rises with neuronal maturation:** MAPT is low in radial glia and TBR2⁺ progenitors, higher in immature TBR1⁺ neurons, and highest in mature SYN1⁺ neurons - **Layer pattern:** Tau mRNA is higher in SVZ and especially cortical plate vs ganglionic eminence - **Confirmation in tissue and organoids:** RNAscope shows weak MAPT in VZ/SVZ-like zones and strong in cortical plate–like regions; organoids increase over time (D35 < D50 < D134) - **Protein lags mRNA:** Little tau protein in germinal zones (IHC/Western), more in white matter/cortical plate → mRNA up before robust protein - **Conserved tau maturation trajectory:** Human fetal brain and organoids share a conserved tau-upregulation trajectory during neuronal maturation—useful as a baseline for disease models |
| Pérez *et al*. (2021). | 26 transcriptionally distinct clusters with five major cell types:   - Three neurons sub-annotations: - Layer I neurons (cluster 18, 21) - Upper-layer neurons (cluster 0, 1, 2, 17) - Dopaminergic neurons (cluster 15) - Progenitors & radial glia (cluster 4, 6, 10, 16, 24) - Astrocytes (cluster 9, 13) - Microglia (cluster 22, 25) - Other glia (cluster 3, 11, 23) | ***PITRM1, ATF4, DDIT3, HSP60, HSPA9, ERO1, LONP1, CLPP, APP*** | - **Broad energy-pathway disruption:** scRNA-seq (26 clusters) shows a similar cell mix across genotypes, but mitochondria/oxidative phosphorylation/sirtuin programs are consistently dysregulated in neurons, glia, astrocytes, and microglia - **Synapse & signalling changes:** Neurons have altered synaptogenesis/LTP; astrocytes show dysregulated iNOS, PPAR, TNFR1, RAR, chemokine, and IL-17A pathways - **Pathology up, partial rescue:** KO organoids ↑APP, ↑Aβ_40/42_ and Aβ_42/40_, and tau hyperphosphorylation. **NMN** lowers Aβ_42/40_ and pTau/total-tau and reduces cleaved caspase-3⁺ cells |
| (Bertacchi *et al*. 2024). | 15 transcriptionally distinct clusters with two major classes:   - Neural progenitors (cluster 2, 5, 8, 9, 12, 13, 14, 15) - Neurons (cluster 1, 3, 4, 6, 7, 10, 11)   Axis/region notes (from sub-analysis):   - Ventral GABAergic identity (cluster 6, 7) - Mixed dorsal/ventral and posterior features (cluster 8, 9, 10, 11) | ***FGF8, FOXG1, OTX2, ASCL1, DLX2, PBX3, EMX1, NEUROG2, SOX5, LHX2, ETV1, NR2F1, FGFR3, ZIC1, ZIC3*** | - **Enhanced regional diversity:** FGF8 boosts brain regional heterogeneity vs WNTi alone - **Coexistence of distinct regional identities:** scRNA-seq finds FOXG1⁺ telencephalic plus OTX2/IRX3/EN1/EN2 non-telencephalic states, IF shows adjacent **FOXG1⁺** and **FOXG1⁻/OTX2⁺** zones - **Diversification of neuronal lineage specification:** Glutamatergic and GABAergic lineages co-exist, with FGF8 promoting antero-ventral patterning |
| (Sarrafha *et al.* 2023). | Broad cell types (12 total; cluster number not specified):   - Neurons - Astrocytes - Radial glial cells - Intermediate progenitor cells - OPCs - Oligodendrocytes - Ependymal cells - Tanycytes - Endothelial cells - VLMCs - Microglia - Unassigned   Neuronal subtypes (17 total; cluster number not specified):   - Neuroendocrine-like cells - Unknown neuron subtype - Unclassified - Dopaminergic neurons - SF1 neurons - SST neurons - GABAergic neurons - Preoptic-area neurons - TH+ GABAergic neurons - Glutamatergic neurons - TRH neurons - MCH neurons - OXT neurons - HCRT neurons - NTS neurons - POMC neurons. - NPW neurons (plus TAC1 and midbrain neurons listed as minor categories) | ***TH, NKX2-1, OTP, POMC, TRH, GFAP, SOX9, SLC17A6 (VGLUT2), GAD2, DLX5, SST, TAC1, HCRT, NR5A1, FOXG1, RAX, DCX, ASCL1, TOP2A, OLIG2, MOBP, CCDC153, COL1A1, DCN, AIF1, DDC, KCNJ6, PRDM12, NR2F2, KCNJ3, NR5A2, TENT5A, CREB3*** | - **Validation of hypothalamic specification and dopaminergic heterogeneity:** D85 organoids contain diverse cell types plus 17 neuronal subtypes; dopaminergic neurons split into **7 subtypes** based on **OTP/RAX** states (four **OTP⁺RAX⁺** groups, **OTP⁺RAX⁻, OTP⁻RAX⁻,** and **KISS1-like),** defined by marker combos (SHH, PRDM12, ASCL1, NR2F2, KCNJ3/KCNJ6) - **Trajectory inference of dopaminergic maturation:** RNA-velocity/pseudotime tracks **OTP⁺RAX⁺ → OTP⁺RAX⁻ / OTP⁻RAX⁻,** with **KISS1-like** neurons the most mature - **Metabolic reprogramming in dopaminergic neurons:** Dopaminergic neurons upregulate **glycolysis, gluconeogenesis, and oxidative phosphorylation**, while **EIF2 signalling, cholesterol biosynthesis, tRNA charging,** and **mevalonate–geranylgeranyl** pathways are down—consistent with higher energy demand |
| (Jain *et al*. 2025). | Major cell types identified (cluster number not specified):   - Early neuroectoderm - Late neuroectoderm - Telencephalic progenitors - Telencephalic neurons - Diencephalic progenitors - Diencephalic neurons - Mesencephalic/Rhomcencephalic progenitors & neurons - Neural crest & peripheral nervous system neurons - Retinal progenitors - Choroid plexus | ***YAP1, WLS, POU5F1, ITGA5, PROM1, THY1, SOX2, TCF7L2, OTX2, ZIC2, CYP26A1, ITGA6, SOX21, COL1A1, COL11A1, LAMA5, HAPLN3, MMP16, IGFBP2, SIX3, LHX2, NRG1, FOXH1, HESX1, RSPO3, GPC3, SFRP2, NPTX1, PRTG, PODXL, RAX, PAX6, SOX10*** | - **Extracellular matrix–dependent regulation of neuroepithelial morphogenesis and regional specification:** Exogenous ECM incorporation (e.g., Matrigel or laminin-PEG) accelerates lumen expansion, promotes neuroepithelial maturation, and biases patterning toward telencephalic identity, whereas absence of ECM or use of agarose results in reduced lumen growth, delayed fusion, and increased neural crest and caudal identities. - **Mechanotransductive control of patterning via YAP–WNT signalling axis**: In the absence of an extrinsic extracellular matrix, YAP1 activity is elevated and directly induces WLS expression, promoting caudalisation of the developing neuroepithelium. Genetic ablation of WLS prevents this shift, restoring telencephalic identity even under matrix-deprived conditions. |
| (Dai *et al*. 2024) | Eight major brain cell classes evaluated (cluster number not specified):   - Excitatory neurons - Inhibitory neurons - Astrocytes - Oligodendrocytes - OPCs - Microglia - Endothelial cells - Pericytes   Note: Organoid benchmarking also referenced radial-glia/neuronal subclasses but without fixed cluster numbers. | ***NRGN, GAD1, CALM2*** | - **Benchmarking of deconvolution methods:** **dtangle** best for cell-type fractions; **bMIND** best for per-sample cell-type expression (ρ ≈ 0.62 ROSMAP, 0.75 CMC, 0.85 organoids). - **Enhanced genetic signal detection through deconvolution:** Mapped **25,273** decon-eQTLs (8 cell types); they explain more SCZ GWAS signal than single cell eQTLs; **decon + bulk ≈ 63%** heritability vs **~49%** with bulk alone. - **Robustness across datasets with reduced sensitivity for rare populations:** AD/SCZ/development DE results replicate in bulk; accuracy drops for **rare cell types (<5%).** - **Recommended analytical framework for brain tissue deconvolution:** Use **dtangle (fractions) + bMIND (expression)** for brain deconvolution. |

**Abbreviations:** Aβ=amyloid beta, AD=Alzheimer’s disease, APOE = apolipoprotein E, APP = amyloid precursor protein, BACE1 = beta-secretase 1, bMIND=Bayesian Model-based INtegration from Deconvolution, CMC=CommonMind Consortium, CO=cerebral organoid, DE=differential expression, DHC=dehydrocholesterol, dtangle=deconvolution method for cell-type heterogeneity, ECM=extracellular matrix, eQTLs=expression quantitative trait loci, fAD=familial Alzheimer’s disease, FGF8 = fibroblast growth factor 8, GWAS=genome-wide association study, hcO-MPSs=human cortical organoid–microphysiological systems, HHV=human herpesvirus, hiPSC=human induced pluripotent stem cell, hPSC=human pluripotent stem cell, HSV-1=herpes simplex virus type 1, IF=immunofluorescence, IHC=immunohistochemistry, KO=knockout, LTP=long-term potentiation, mhCOs=microglia-containing human cortical organoids, mRNA=messenger RNA, NDC=non-demented control, NFTs=neurofibrillary tangles, NMN=nicotinamide mononucleotide, NSCs=neural stem cells, OC=occipital cortex, oMG= organoid-derived microglia-like cells , OPCs=oligodendrocyte precursor cells, OTC=occipitotemporal cortex, PFASs=per- and polyfluoroalkyl substances, PHF-tau=paired helical filament tau, pTau=phosphorylated tau, PSEN1 = presenilin 1, PSEN2 = presenilin 2, ROSMAP=Religious Orders Study and Memory and Aging Project, scRNA-seq=single cell RNA sequencing, SCZ=schizophrenia, SVZ=subventricular zone, TEs=transposable elements, TFs=transcription factors, VLMCs=vascular and leptomeningeal cells, VZ=ventricular zone, WNTi=WNT inhibition

**References:**

Cakir, B., Tanaka, Y., Kiral, F. R., Xiang, Y., Dagliyan, O., Wang, J., Lee, M., Greaney, A. M., Yang, W. S., duBoulay, C., Kural, M. H., Patterson, B., Zhong, M., Kim, J., Bai, Y., Min, W., Niklason, L. E., Patra, P., & Park, I.-H. (2022). Expression of the transcription factor PU.1 induces the generation of microglia-like cells in human cortical organoids. *Nature Communications*, *13*(1), 430. https://doi.org/10.1038/s41467-022-28043-y

Chen, X., Sun, G., Tian, E., Zhang, M., Davtyan, H., Beach, T. G., Reiman, E. M., Blurton-Jones, M., Holtzman, D. M., & Shi, Y. (2021). Modeling Sporadic Alzheimer’s Disease in Human Brain Organoids under Serum Exposure. *Advanced Science*, *8*(18), 2101462. https://doi.org/10.1002/advs.202101462

Choe, M. S., Yeo, H. C., Kim, J. S., Lee, J., Lee, H. J., Kim, H.-R., Baek, K. M., Jung, N.-Y., Choi, M., & Lee, M. Y. (2024). Simple modeling of familial Alzheimer’s disease using human pluripotent stem cell-derived cerebral organoid technology. *Stem Cell Research & Therapy*, *15*(1), 118. https://doi.org/10.1186/s13287-024-03732-1

Feng, Y., Cao, S.-Q., Shi, Y., Sun, A., Flanagan, M. E., Leverenz, J. B., Pieper, A. A., Jung, J. U., Cummings, J., Fang, E. F., Zhang, P., & Cheng, F. (2025). Human herpesvirus-associated transposable element activation in human aging brains with Alzheimer’s disease. *Alzheimer’s & Dementia*, *21*(2), e14595. https://doi.org/10.1002/alz.14595

Fiock, K. L., Smalley, M. E., Crary, J. F., Pasca, A. M., & Hefti, M. M. (2020). Increased Tau Expression Correlates with Neuronal Maturation in the Developing Human Cerebral Cortex. *eNeuro*, *7*(3), ENEURO.0058-20.2020. https://doi.org/10.1523/ENEURO.0058-20.2020

Glasauer, S. M. K., Goderie, S. K., Rauch, J. N., Guzman, E., Audouard, M., Bertucci, T., Joy, S., Rommelfanger, E., Luna, G., Keane-Rivera, E., Lotz, S., Borden, S., Armando, A. M., Quehenberger, O., Temple, S., & Kosik, K. S. (2022). Human tau mutations in cerebral organoids induce a progressive dyshomeostasis of cholesterol. *Stem Cell Reports*, *17*(9), 2127–2140. https://doi.org/10.1016/j.stemcr.2022.07.011

Lu, S., Zhu, X., Zeng, P., Hu, L., Huang, Y., Guo, X., Chen, Q., Wang, Y., Lai, L., Xue, A., Wang, Y., Wang, Z., Song, W., Liu, Q., Bian, G., Li, J., Bu, Q., & Cen, X. (2024). Exposure to PFOA, PFOS, and PFHxS induces Alzheimer’s disease-like neuropathology in cerebral organoids. *Environmental Pollution (Barking, Essex: 1987)*, *363*(Pt 1), 125098. https://doi.org/10.1016/j.envpol.2024.125098

Perez-Corredor, P., Vanderleest, T. E., Vacano, G. N., Sanchez, J. S., Villalba-Moreno, N. D., Marino, C., Krasemann, S., Mendivil-Perez, M. A., Aguillón, D., Jiménez-Del-Río, M., Baena, A., Sepulveda-Falla, D., Lopera, F., Quiroz, Y. T., Arboleda-Velasquez, J. F., & Mazzarino, R. C. (2024). APOE3 Christchurch modulates β-catenin/Wnt signaling in iPS cell-derived cerebral organoids from Alzheimer’s cases. *Frontiers in Molecular Neuroscience*, *17*. https://doi.org/10.3389/fnmol.2024.1373568

Tian, C., Ao, Z., Cerneckis, J., Cai, H., Chen, L., Niu, H., Takayama, K., Kim, J., Shi, Y., Gu, M., Kanekiyo, T., & Guo, F. (2025). Understanding monocyte-driven neuroinflammation in Alzheimer’s disease using human cortical organoid microphysiological systems. *Science Advances*, *11*(34), eadu2708. https://doi.org/10.1126/sciadv.adu2708

Vanova, T., Sedmik, J., Raska, J., Cerna, K. A., Taus, P., Pospisilova, V., Nezvedova, M., Fedorova, V., Kadakova, S., Klimova, H., Capandova, M., Orviska, P., Fojtik, P., Bartova, S., Plevova, K., Spacil, Z., Hribkova, H., & Bohaciakova, D. (2023). Cerebral organoids derived from patients with Alzheimer’s disease with PSEN1/2 mutations have defective tissue patterning and altered development. *Cell Reports*, *42*(11). https://doi.org/10.1016/j.celrep.2023.113310

Zhao, J., Ikezu, T. C., Lu, W., Macyczko, J. R., Li, Y., Lewis-Tuffin, L. J., Martens, Y. A., Ren, Y., Zhu, Y., Asmann, Y. W., Ertekin-Taner, N., Kanekiyo, T., & Bu, G. (2023). APOE deficiency impacts neural differentiation and cholesterol biosynthesis in human iPSC-derived cerebral organoids. *Stem Cell Research & Therapy*, *14*, 214. https://doi.org/10.1186/s13287-023-03444-y
